# Retinal pigment epithelial cells secrete neurotrophic factors and synthesize dopamine: possible contribution to therapeutic effects of RPE cell transplantation in Parkinson's disease

**DOI:** 10.1186/1479-5876-7-53

**Published:** 2009-06-28

**Authors:** Ming Ming, Xuping Li, Xiaolan Fan, Dehua Yang, Liang Li, Sheng Chen, Qing Gu, Weidong Le

**Affiliations:** 1 Institute of Health Sciences, Shanghai Institutes for Biological Sciences, Chinese Academy of Sciences, and Shanghai Jiao Tong University School of Medicine, Shanghai, 200025, PR China; 2 Institute of Neurology, Ruijin Hospital, Jiao Tong University School of Medicine, Shanghai, 200025, PR China; 3 Department of Ophthalmology, Shanghai First People's Hospital, Shanghai, 200025, PR China

## Abstract

**Background:**

New strategies for the treatment of Parkinson's disease (PD) are shifted from dopamine (DA) replacement to regeneration or restoration of the nigro-striatal system. A cell therapy using human retinal pigment epithelial (RPE) cells as substitution for degenerated dopaminergic (DAergic) neurons has been developed and showed promising prospect in clinical treatment of PD, but the exact mechanism underlying this therapy is not fully elucidated. In the present study, we investigated whether the beneficial effects of this therapy are related to the trophic properties of RPE cells and their ability to synthesize DA.

**Methods:**

We evaluated the protective effects of conditioned medium (CM) from cultured RPE cells on the DAergic cells against 6-hydroxydopamine (6-OHDA)- and rotenone-induced neurotoxicity and determined the levels of glial cell derived neurotrophic factor (GDNF) and brain derived neurotrophic factor (BDNF) released by RPE cells. We also measured the DA synthesis and release. Finally we transplanted microcarriers-RPE cells into 6-OHDA lesioned rats and observed the improvement in apomorphine-induced rotations (AIR).

**Results:**

We report here: (1) CM from RPE cells can secret trophic factors GDNF and BDNF, and protect DAergic neurons against the 6-OHDA- and rotenone-induced cell injury; (2) cultured RPE cells express L-dopa decarboxylase (DDC) and synthesize DA; (3) RPE cells attached to microcarriers can survive in the host striatum and improve the AIR in 6-OHDA-lesioned animal model of PD; (4) GDNF and BDNF levels are found significantly higher in the RPE cell-grafted tissues.

**Conclusion:**

These findings indicate the RPE cells have the ability to secret GDNF and BDNF, and synthesize DA, which probably contribute to the therapeutic effects of RPE cell transplantation in PD.

## Background

Parkinson's disease is a neurodegenerative disorder which affects approximately 1% population over the age of 60 [1]. The most motor symptoms of this disease are caused by dysfunction of the nigro-striatal pathway. DAergic neurons in the substantial nigral pars compacta (SNc) project axons to striatum; when PD patients display symptoms, more than half of the DAergic neurons in the SNc are lost. In the last two decades, several different sources of DAergic cells as transplantation therapy have been tried in animal models and in patients with PD [2-6]. RPE cell transplantation has been applied in experimental and clinical studies for its capability of producing L-dopa as intermediate product of melanin [7,8]. RPE cell transplantation therapy has many advantages: it does not require immune suppression, the cells are relatively easy to obtain, and the procedure has minimal ethic concern, which make this approach attractive [9].

RPE cells are melanin containing cells that constitute a monolayer between the neural retina and the choroid. In RPE cells, tyrosine is catalyzed by tyrosinase to L-dopa that is polymerized to form melanin [10]. It is hypothesized that L-dopa in the RPE cells can be converted into DA in the terminates of nigrostriatal DAergic neurons and provide DA to nigro-striatal system directly after RPE cells are transplanted [7]. However, such assumption has yet to be verified.

RPE cells play a key role in maintaining the normal function of retina and can express several neurotrophic factors such as platelet-derived growth factor (PDGF), epidermal growth factor (EGF), vascular endothelial growth factor (VEGF), and pigment-derived epithelial factor (PEDF) [11], which nourish the neurosensory retina and also probably provide trophic effects on the host DAergic neurons.

In the present study, we attempt to determine whether the neurotrophic effects of RPE cells play a role in restoring the function of nigrostriatal system in the transplanted model of PD, and to examine whether RPE cells have the ability to synthesize and release DA in the cultures. Our works provide the first evidence that RPE cells can secrete the neurotrophic factors GDNF and BDNF, and synthesize DA, which probably contribute to their beneficial effects of RPE cells transplantation in animal model of PD.

## Methods

### Cell cultures

Human RPE cells were obtained from the RPE Cell Bank at the Shanghai 1^st ^Hospital. The method to collect the RPE cells was similar to the previous description [12]. In brief, human eyes were dissected by a circumferential incision above the ora serrata near the limbus; the anterior segment and lens were separated and discarded. The neural retina was detached and layer of RPE cells were separated from the choroid. The layer of RPE cells was dissociated in 0.25% trypsin (Gibco-Invitrogen, USA), by gentle titration, and the cells were collected by centrifuge at 100 × g for 5 minutes. Then the cells were calculated and seeded at the density of 10^5 ^per cm^2^. Growing medium consisted of Dulbecco's modified Eagle's medium (DMEM, Gibco-Invitrogen, USA), 10% fetal bovine serum (FBS, heat-inactivated, Gibco-Invitrogen, USA) and 100 unit/ml penicillin and streptomycin. At confluence, cells were subcultured by trypsinization.

SH-SY5Y cells were cultured on poly-D-lysine (Sigma, USA) precoated dishes in DMEM supplemented with 10% FBS, and the medium was changed every 3 days.

To culture primary ventral mesencephalic (VM) cells, pregnant Sprague-Dawley (SD) rats at gestation day 14 (Experimental Animal Center of Shanghai, China) were anaesthetized with chloral hydrate (400 mg/kg, i.p.) and VM tissues were dissected from embryonic brain and trypsinized into single-cell suspension using sterilized micropipette tips. The cells were resuspended in DMEM and Ham's F12 at 1:1 (D-F12), supplemented with 10% FBS and plated at a final density of 5 × 10^5 ^viable cells/cm^2 ^in 24-well plates (Nunc, Denmark) precoated with poly-D-lysine. The cells were incubated at 37°C for 12 hours and then switched to the serum-free medium, consisting of D-F12 with 2% B27 supplement (Gibco-Invitrogen, USA). For differentiation of VM cells, the cultured cells were incubated in serum-free medium for 6 days, and the culture medium was changed each 3 days.

### Preparation of conditioned medium

CM by RPE cells (RPE-CM) was collected as previously described [13]. Briefly, RPE cells were incubated with FBS-deprived medium for 3 days, and the medium was collected and centrifuged at 1000 × *g *for 10 minutes at 4°C to remove cells and debris. The supernatant was concentrated 5-fold in an Amicon Ultra tube (Millipore, USA) by centrifugation (4,000 × *g*, 2 hours) at 4°C. The concentrated medium was diluted by fresh DMEM to 1-fold concentration. The proteins which molecular weight is lower than 10 kDa were removed by filtration.

### Determine the protective role of RPE cells in vitro

SH-SY5Y cells were incubated in DMEM supplemented with 10% FBS. Then the culture medium were replaced with three different medium. One group was incubated with the RPE-CM containing rotenone or 6-OHDA. The second group was exposed to the normal medium containing rotenone or 6-OHDA. The third group was cultured in normal medium without toxins. After 24 hours of incubation, 10 μl of the dye 3, [4,5-dimethylthiazol-2-yl]-diphenyltetrazolium bromide (MTT) (5 mg/ml) was added to make the final concentration at 0.5 mg/ml, and then the plates were incubated for 4 hours at 37°C. After medium were removed, 100 μl dimethyl sulfoxide per well was added and the plate was incubated at 37°C for 15 minutes. Color intensity was assessed with a microplate reader at the 570 nm wavelength. Each experiment was performed in triplicate independently.

In order to test the protective role of CM against rotenone, the VM cultures were treated under different conditions as following for 8 hours. 1) RPE-CM with 25 nM rotenone; 2) normal medium with 25 nM rotenone; 3) normal medium without rotenone. Then the neurons were immunostained against TH and the number of TH-immunoreactive (TH-ir) neurons was counted in a blind manner by an unrelated investigator. Ten fields per well (113 mm^2 ^surface area) were counted using a field lens, and the size of field was 4 mm^2 ^and 10 fields consisted of about 35% of the whole surface of the cultured well.

For 6-OHDA treatment, the VM cultures were treated under different conditions as following for 24 hours: 1) RPE-CM with 40 μM 6-OHDA; 2) normal medium with 40 μM 6-OHDA; 3) normal medium without 6-OHDA.

### Measurement of GDNF and BDNF using Enzyme-linked immunosorbent assay (ELISA)

After two days culture of 10^6 ^RPE cells, 2 ml serum-free medium were collected and ultrafiltered using the Amicon Ultra Tube (10 kDa). To determine in vivo neurotrophic factors expression, 15 mg wet tissues with microcarriers-RPE cells and tissues with microcarriers were lysed for ELISA assay. The lysis buffer was prepared according to the manual in a ratio of 1 mg tissue to 10 μl buffer. The concentrations of GDNF and BDNF were determined using Emax ImmunoAssay System (Promega, USA) [14].

### High performance liquid chromatography (HPLC) analysis

10^6 ^RPE cells were homogenized in 200 μl 0.4 M perchloric acid. Homogenates were centrifuged at 12,000 rpm for 20 minutes at 4°C and the supernatants were collected for HPLC (Eicom HTEC-500, Japan), while the pellet was dissolved in 0.1 M NaOH for BCA protein analysis (Pierce, USA). One liter mobile phage was consisted of 8.84 g citric acid monohydrate, 10 g sodium acetate anhydrate, 220 mg sodium octane sulfonate, 5 mg EDTA-2Na and 200 ml methanol.

To analyze the DA release, the RPE cells was treated with high potassium solution (56 mM K^+^) (84 mM NaCl, 55 mM KCl, 1 mM MgSO_4_, 1.25 mM KH_2_PO_4_, 2 mM CaCl_2_, 16 mM NaHCO_3_, and 10 mM glucose) as previously described [15]. The high potassium solution collected from RPE cells was mixed with 0.4 M perchloric acid in the ratio of 1:1 and was centrifuged before HPLC assay.

### Western blot

10^6 ^RPE cells were lysed in RIPA lysis buffer [(in mM): Tris-HCl, 50, pH 7.4; NaCl, 150; 0.1% sodium dodecyl sulphate (SDS), EDTA, 1; 1% Triton X-100, 1% sodium deoxycholate, PMSF, 1; 5 μg/ml aprotinin, 5 μg/ml leupeptin]. Protein concentration was measured and 40 μg of total proteins were loaded to SDS-polyacrylamide gel electrophoresis (SDS-PAGE). The separated proteins were transferred onto polyvinylidene difluoride (PVDF, Millipore, USA) membrane, and incubated with anti-DDC antibody (Proteintech Group, USA) or anti-dopamine transporter (DAT) antibody (Santa Cruz, USA) overnight. After incubation, the membrane was washed and incubated with peroxidase-conjugated goat anti-rabbit IgG (Pierce, USA), and developed with Super Signal West Dura Extended Duration Substrate (Pierce, USA).

### Reverse transcription PCR analysis

Total RNA from RPE cells was prepared using Trizol reagent (Invitrogen, USA) and digested with RNase-free DNase for 30 minutes to remove genomic DNA. 2 μg of RNA were reverse transcribed into cDNA with the Reverse Transcription System (Promega, USA) in 20 μl volume. cDNA was used as template in the following PCR assay. The primers used for PCR assays were as follows: (1) DDC, forward: 5'-TTACTCATCCGATCAGGCACAC-3', reverse: 5'-GGCAGAACAGTCAAAATTCACC-3'; (2) DAT, forward: 5'-CGAGGCGTCTGTTTGGAT-3', reverse: 5'-CAGGGAGTTGATGGAGGTG-3'; (3) GAPDH, forward: 5'-CCATGTTCGTCATGGGTGTGAACCA-3', reverse: 5'-GCCAGTAGAGGCAGGGATGATGTTC-3'. PCR conditions were 95°C for 10 minutes, followed by 35 cycles of 94°C for 45 seconds, 58°C for 45 seconds, 72°C for 1 minute, and a final extension step at 72°C for 5 minutes.

### RPE cells-microcarriers attachment

The microcarriers which were dextran particles coated with gelatin (Cytodex 3, Sigma, USA) were sterilized and hydrated according to the manufacture manual (Sigma, USA). Dry microcarriers were swollen in Ca^2+^, Mg^2+^-free phosphate buffered saline (PBS) (50–100 ml/g Cytodex) for at least 3 hours and the microcarriers were sterilized by autoclaving (120°C, 20 minutes). The microcarriers were rinsed using medium three times before mixed with RPE cells, suspended at 2 × 10^6 ^density in 1 ml medium and then mixed with 10^5 ^microcarriers. The mixture was shaken in the rate of 60 rpm for 2 hours at 37°C, and then was cultured for 24 hours [16].

### 6-OHDA lesion and RPE cell transplantation

SD rats were housed pre- and post-surgery in a temperature and humidity controlled room with a 12 hours light-dark cycle. Food and water were freely available.

Experimental rats were anesthetized with chloral hydrate (400 mg/kg) and received brain injection on a stereotaxic frame (Myneurolab, USA). 6-OHDA (6 μg in 3 μl) was dissolved in normal saline containing ascorbic acid (0.2 mg/ml), and injected into the right medial forebrain bundles (MFB; anterior-posterior: -4.2 mm, lateral: -1.5 mm from bregma, dorsal-ventral: -7.7 mm from dura, toothbar set at -2.4 mm) via a 10 μl hamilton syringe with a blunt-tip needle at a flow rate of 1 μl/minute. After injection, the needle was left *in situ *for 10 minutes and then slowly withdrawn. A gelfoam plug was placed on the broken dura and the skin was sutured [17].

Microcarriers with RPE cells were washed three times with Ca^2+^, Mg^2+^-free PBS and were kept at 4°C before transplantation. The cell transplantation was performed by stereotaxic injection into the right side of striatum (anterior-posterior 1.5 mm, lateral -2.0 mm from bregma, dorsal-ventral -5.0 mm from dura, set toothbar at -3.3 mm) as described previously [18]. Some of the rats were transplanted with the microcarriers alone as control.

### Behavioural testing

SD rats were tested for the AIR behavior two weeks after 6-OHDA lesion and four weeks after transplantation by administration with apomorphine (0.2 mg/kg, i.p.). Only the rats that exhibited a mean rotation toward the healthy side at least 6.0 full body turns per minute were used for transplantation. Four weeks after transplantation, rotation behavior of rats was examined again. Rats transplanted with microcarriers alone were used as control for the behavior test.

### Histological procedure and immunostaining

Rats were deeply anesthetized with chloral hydrate and sacrificed by transcardial perfusion with PBS (37°C) for 20 minutes followed by 4% paraformaldehyde (PFA) (4°C) for 10 minutes. Brains were removed, postfixed for 2 hours in PFA and then cryoprotected for 24 hours in PBS with 30% sucrose. Before frozen in -80°C, the brains were embedded in embedding medium compound (Sakura, USA). Coronal sections (10 μm) were made through the striatum containing transplants and mounted to gelatin coated slides. Adjacent sections were processed for hematoxylin-eosin (HE) stain and immunohistochemistry.

The sections were stained against cytokeratin antibody (1:300, Sigma, USA), and the primary VM neurons were stained against TH (1:3000, sigma, USA). A biotinylated secondary rabbit anti-mouse antibody (Vector Laboratories, UK) and peroxidase-coupled avidin-biotin staining kit (ABC kit, Vector Laboratories, UK) were used.

For HE staining, the tissue sections were submerged into the hematoxylin solution (0.5% hematoxylin, 5% aluminium ammonium sulphate, 1% ethanol, 0.1% sodium iodate, 2% acetic acid and 30% glycerol) for 10 minutes and washed by tap water. Place the sections in acid alcohol (0.3% concentrated hydrochloric acid in 70% ethanol) for several seconds and then in eosin solution (0.1% eosin, 0.4% acetic acid in 95% ethanol) for 1 minute. Then the sections were dehydrated and sealed.

### Statistics

All data were expressed as means ± SEM. Independent *t*-test followed by *post hoc *Bonferroni tests were used for the analysis of other data via the SPSS 10.0 soft packages (SPSS Inc., USA). The criterion for statistical significance was set at *p *< 0.05.

## Results

### RPE-CM protects against rotenone and 6-OHDA toxicity through GDNF and BDNF secretion

**Figure 1:**
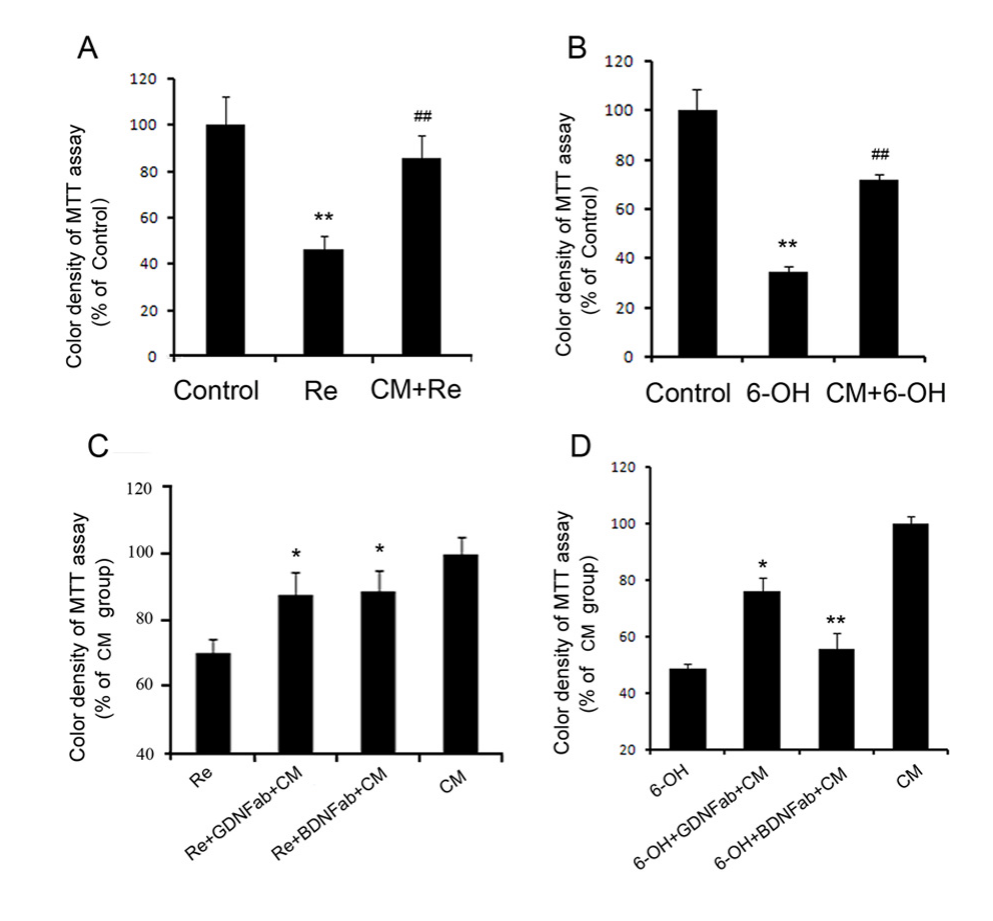
**RPE-CM protect SH-SY5Y cells from injury in the presence of neurotoxins**. (A) SH-SY5Y cells cultured without rotenone (Control), cells treated with 10 μM rotenone (Re), cells treated with 10 μM rotenone in RPE-CM (CM+Re) were examined by MTT assay. Rotenone treatment produce significant cell lose in SH-SY5Y cultures (**p < 0.01 compared with control). RPE-CM significantly attenuated rotenone-induced cell loss (##p < 0.01 compared with rotenone group). (B) SH-SY5Y cells were treated as in A in the presence of 50 μM 6-OHDA. 6-OHDA treatment produced significant cell lose in SH-SY5Y cultures (**p < 0.01 compared with CM treated control). RPE-CM significantly attenuated 6-OHDA-induced cell loss (## p < 0.01 compared with 6-OHDA treated group). (C) Blockage of GDNF and BDNF by antibodies inhibited the protection of the RPE-CM. RPE-CM was pretreated with 1 μg/ml GDNF antibody (Re+GDNFab+CM) or with 1 μg/ml BDNF antibody (Re+BDNFab+CM) and incubated with SH-SY5Y cells in the presence of 10 μM rotenone. The protective effect of RPE-CM could be partially blocked by GDNF and BDNF antibodies (*p < 0.05 compared with CM treated group). (D) Cells were treated as in C in the presence of 50 μM 6-OHDA. The protective effect of RPE-CM could be partially blocked by GDNF and BDNF antibodies when treated with 6-OHDA (*p < 0.05 compared with CM treated group; **p < 0.01 compared with CM treated control). Data showed the mean ± SEM values from three independent experiments performed in triplicate.

The neuroprotective ability of the RPE-CM was determined by adding CM into neurotoxins-treated DAergic cell cultures. SH-SY5Y cultures were challenged by rotenone or 6-OHDA. After exposure to 10 μM rotenone for 24 hours, the cell viability in the cultures was determined by MTT assay. It was found that rotenone treatment resulted in 43.1% decrease in cell viability as compared with control cultures (Fig 1A). Incubation with RPE-CM significantly attenuated the rotenone-induced decrease in cell viability by 72.9% (Fig 1A). When SH-SY5Y cells were challenged by 50 μM 6-OHDA, RPE-CM showed a similar protective ability on the cells viability. 6-OHDA decreased the cell viability by 65.3%, treatment with RPE-CM protected the cell viability by 56.7% (Fig 1B).

BDNF and GDNF are believed to be the most important neurotrophic factors in the survival of DAergic cells [14,19,20]. So we focused on these two neurotrophic factors and measured the level of these two neurotrophic factors by ELISA assay to determine whether the protective effect of RPE-CM is mediated by the secretion of GDNF and BDNF. We found the RPE-CM contained high levels of GDNF (0.019 pg/ml) and BDNF (0.49 pg/ml) (Table 1). Furthermore, adding antibodies of GDNF and BDNF to abolish their biological effects demonstrated that GDNF and BDNF were key elements in the neurotrophic protection of RPE-CM. RPE-CM with antibodies against GDNF or BDNF (1 μg/ml) was added into the SH-SY5Y cultures in the presence of 10 μM rotenone or 50 μM 6-OHDA. After 24 hours incubation, the cell viability of SH-SH5Y cultures was measured by MTT assay. Application of antibody against GDNF decreased the CM-mediated protection on SH-SY5Y cells by 41.4% and 46.7% against rotenone and 6-OHDA induced injury, respectively (Fig 1C, D). While antibody against BDNF could reduce the CM-induced protection on SH-SY5Y cells by 38.7% and 85.9% against rotenone and 6-OHDA induced injury, respectively (Fig 1C, D).

**Table 1:** Neurotrophic factors secreted by RPE cells

Trophic factors	BDNF	GDNF
Concentration in medium (pg/ml)	0.49 ± 0.09	0.019 ± 0.005

Serum-free medium was incubated with RPE cells for two days, and subjected to ELISA assay.

**Figure 2:**
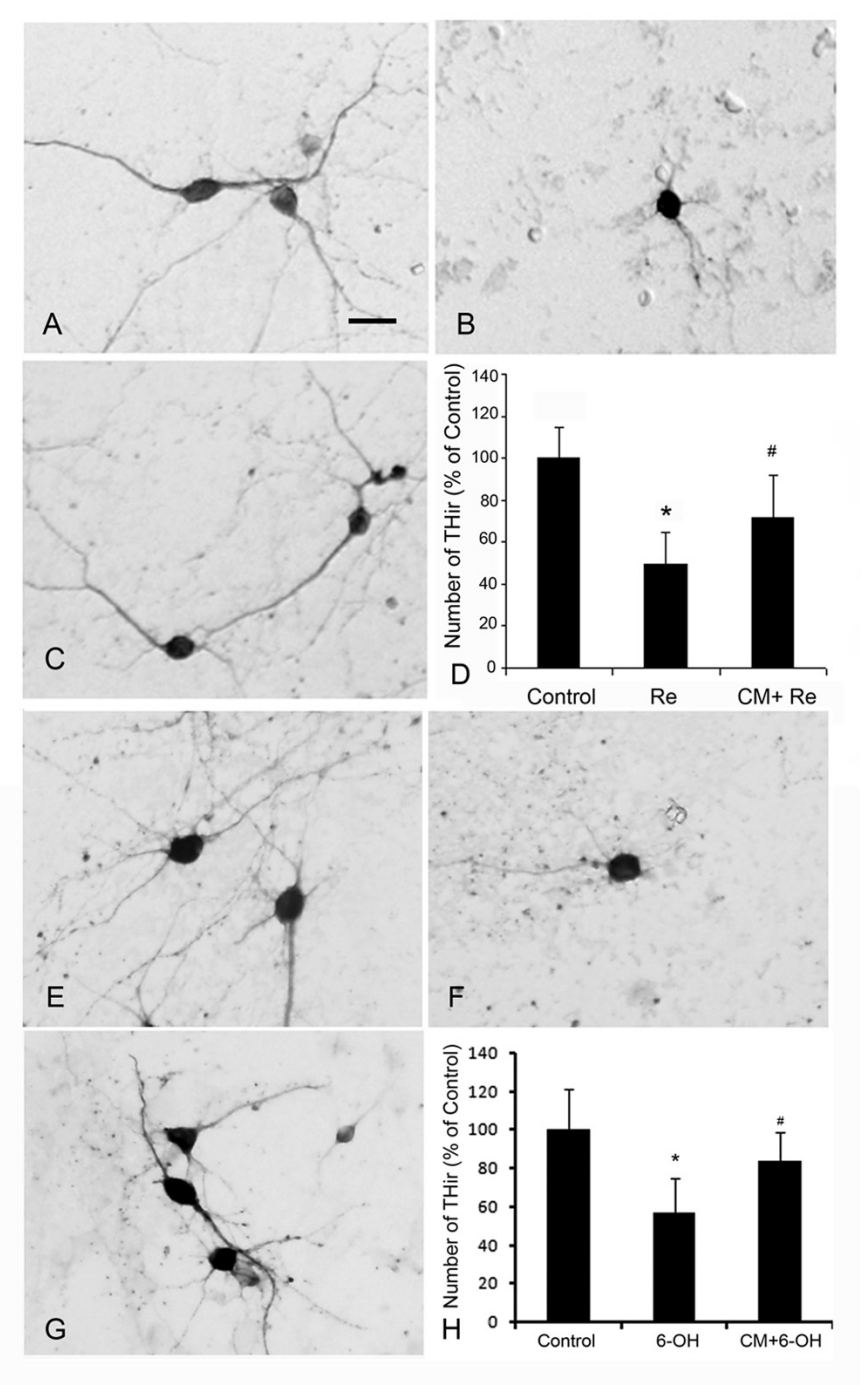
**RPE-CM protects the DAergic neurons against the rotenone- and 6-OHDA- induced neuron loss in primary VM cultures**. (A) VM neurons treated with CM in the presence of 25 nM rotenone. Scale bar, 10 μm. (B) VM neurons cultured in fresh medium in the presence of rotenone. (C) VM neurons cultured in fresh medium without rotenone. (D) The number of TH-ir neurons in the cultures treated with fresh medium only (Control), with fresh medium in the presence of 25 nM rotenone (Re) and with CM in the presence of 25 nM rotenone (CM+ Re). Data represent the mean ± SEM. *p < 0.05. (E) VM neurons treated with CM in the presence of 40 μM 6-OHDA. (F) VM neurons cultured in fresh medium in the presence of 40 μM 6-OHDA. (G) VM neurons cultured in fresh medium without 6-OHDA. (H) The number of TH-ir neurons in the cultures treated with fresh medium (Control), with fresh medium in the presence of 40 μM 6-OHDA (6-OH) and with CM in the presence of 40 μM 6-OHDA (CM+6-OH). Data represent the mean ± SEM. *p < 0.05.

To further support our findings, we then tested the neuroprotection of RPE cells in primary VM DA neurons culture. Exposure to 25 nM rotenone for 8 hours resulted in a significant loss of the TH-positive cells by 50.6% as compared with control cultures without rotenone treatment (Fig 2B), while incubation with RPE-CM significantly attenuated the rotenone-induced loss of TH-positive cells by 44.3% (Fig 2A).

When DAergic neuron cultures were challenged by 6-OHDA at 40 μM, RPE-CM showed a similar protective ability on the TH-positive cells. 6-OHDA treatment caused a 43.2% loss of TH-positive cells as compared with non-toxin control cultures (Fig 2F), while RPE-CM attenuated the 6-OHDA-induced TH-positive cell loss by 63.1% (Fig 2E).

### RPE cells express GDNF and BDNF after transplantation

**Figure 3:**
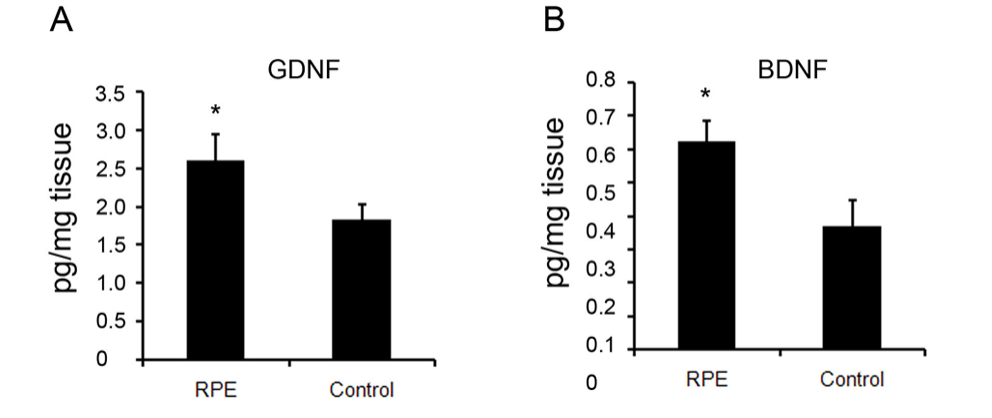
**Determination of GDNF and BDNF from RPE cells after transplantation**. (A) Tissues with transplanted RPE cells were lysed for GDNF determination and tissues with transplanted microcarriers were used as control. (B) Tissues with transplanted RPE cells were lysed for BDNF determination and tissues with transplanted microcarriers were used as control. The concentrations of GDNF and BDNF were determined using Emax ImmunoAssay System (Promega, USA).

As the role of GDNF and BDNF was demonstrated in the neuroprotection of RPE-CM against 6-OHDA and rotenone neurotoxicity in vitro, we measured the levels of GDNF and BDNF in the RPE cell-grafted striatal tissues. Four weeks after transplantation the striatal tissues with microcarriers-RPE cells were taken out and homogenated, followed by centrifugation at 12000 rpm for 20 minutes. The striatal tissues transplanted with microcarriers were used as control. ELISA assay showed that tissues with microcarriers-RPE cells had 41.2% and 68.1% higher levels of GDNF and BDNF as compared with the control group which contained microcarriers only (Fig 3A, B).

### RPE cells express DDC and synthesize DA

**Figure 4:**
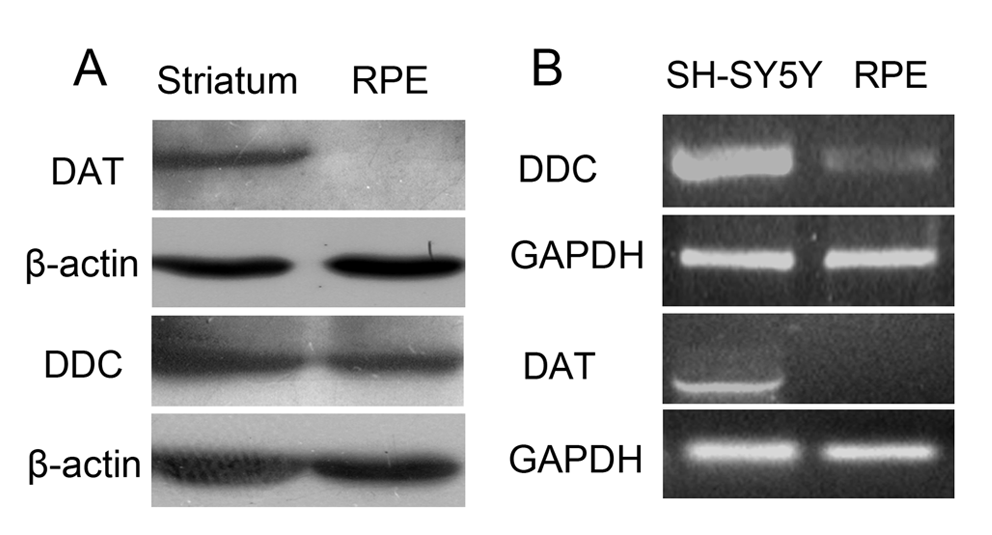
**RPE cells express DDC but not DAT**. (A) DDC from RPE cells was detected by western blot. RPE protein was loaded and the equal level of C57 mouse striatum protein was used as positive control; protein DDC was detected by western blot and both samples displayed the same size protein bands. The protein DAT could not be detected in RPE cells. (B) The cDNA of DDC but not DAT was detected by PCR from the total cDNA of RPE cells.

DDC is an enzyme that converts L-dopa to DA; the expression of DDC indicates the RPE cells have the ability to produce DA. To determine whether the RPE cells can synthesize DA, we measured the DDC mRNA by RT-PCR and DDC protein by immunoblot, which showed that the RPE cells could transcribe DDC mRNA and express abundant DDC protein (Fig 4). But the mRNA and the protein of DAT which transports DA through the membrane couldn't be detected by RT-PCT and immunoblot (Fig 4).

**Figure 5:**
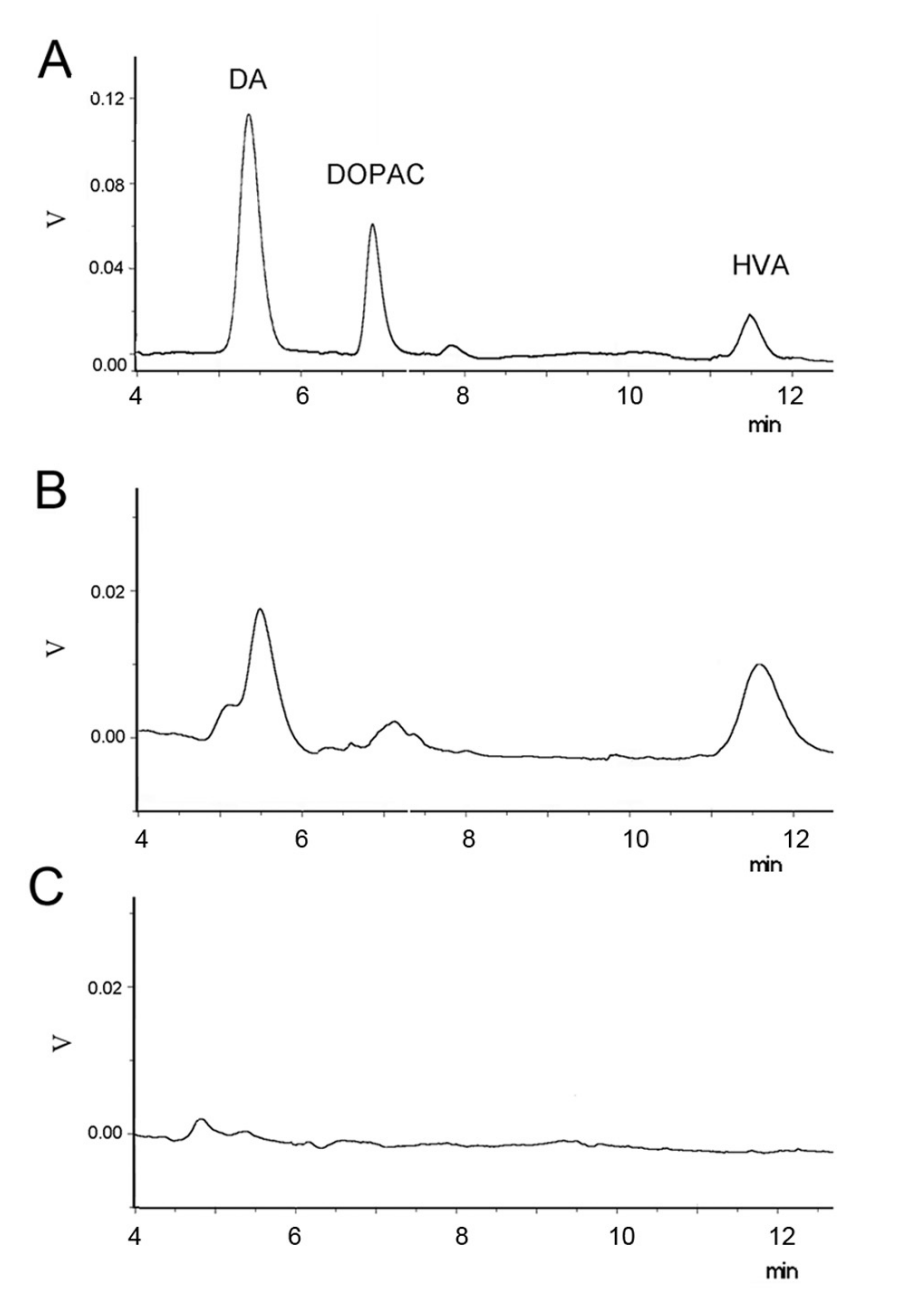
**HPLC analysis of the synthesis and release of DA by RPE cells**.(A) HPLC analysis of standard of DA, 3,4-dihydroxyphenylacetic acid (DOPAC) and HVA. (B) HPLC analysis of RPE cells homogenate. The peaks of DA and HVA in the RPE cells were detected but the DOPAC signal was weak. (C) HPLC analysis of high potassium solution incubated with RPE cells.

Furthermore, we measured the content of DA and its metabolite homovanillic acid (HVA) in RPE cells by HPLC (Fig 5), which showed DA (peak time: 5.43 minutes) level as 29.13 ng/mg protein and HVA (peak time: 11.51 minutes) level as 267.89 ng/mg protein (Table 2). However, DA release into the buffer was not detected after 56 mM potassium chloride treatment in the cultured RPE cells, suggesting that the high potassium-depolarization can not induce DA release from RPE cells (Table 2). It's likely that RPE cells may have other mechanism to transfer DA throughout the membrane.

**Table 2:** DA and HVA in RPE cells extract and DA release after potassium treatment

	DA	HVA
Cells extract (ng/mg protein)	29.13 ± 4.11	267.89 ± 16.10
Release after potassium treatment	None	None

RPE cells were homogenated and centrifuged. The supernatant was examined by HPLC for DA and its metabolic. To determine the DA release, RPE cells were depolarized with high potassium (56 mM K^+^) and the buffer was subjected to HPLC assay.

### Microcarriers-RPE cells survive in the host striatal tissues and significantly improve AIR in 6-OHDA-lesioned rats

**Figure 6:**
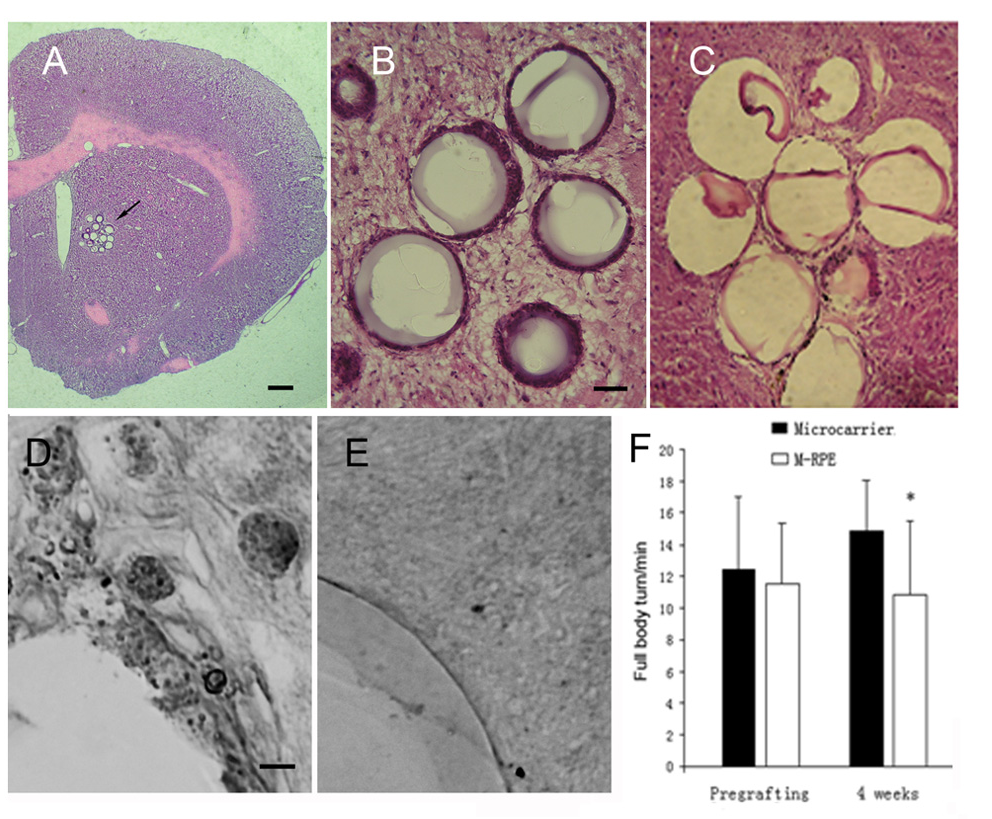
**Microcarriers-RPE cells survive in the host striatum and transplantation with RPE cells significantly improve animal behaviours**. (A) Low magnification microphotogragh of the striatum with transplants. Arrow indicates the transplants. The scale bar is 0.5 mm. (B) HE staining of the corpus striatum of SD rat injected with microcarriers-RPE (M-RPE). 10 μm sections were stained with hematoxylin-eosin. The scale bar is 20 μm. (C) The corpus striatum of SD rat injected with microcarriers alone as control. Sections were stained as in B. (D) Corpus striatum of SD rat injected with M-RPE was immunostained with cytokeratin antibody. The scale bar is 5 μm. (E) The corpus striatum of SD rat injected with microcarriers only was stained as in D. (F) AIR before transplantation and 4 weeks after transplantation. Data are shown as mean ± S.E.M. *p < 0.05. N = 8.

To demonstrate the RPE cells survival in the host striatum, we performed HE staining and cytokeratin immunostaining. HE staining showed that transplants were accurately placed into the striatum (Fig 6A) and RPE cells were attached outside the microcarriers (Fig 6B); immunostaining demonstrated that these cells were cytokeratin-immunoreactive, a morphological marker of live RPE cells (Fig 6D).

Before transplantation, AIR showed the basal level of rotation in 6-OHDA lesioned rats. We selected the rats that exhibited rotation toward the healthy side at least 6.0 full body turns per minute for transplantation of microcarriers-RPE or microcarriers alone as control. After transplantation, microcarriers-RPE grafted animals displayed a significant reduction in AIR behavior compared to control rats that was transplanted with microcarriers alone (*p *< 0.05) (Fig 6F).

## Discussion

Most of the cell-based therapies for PD are focused on two goals: one is to provide a source of neurotrophic factors which may modify disease course and the other is to provide a constant level of DA. RPE cell transplantation is a promising therapy as shown in preliminary clinical trial. In the present study, we attempt to elucidate the mechanisms of this therapy by determining: whether RPE cells exert protective effects on DAergic cells when challenged by neurotoxins; whether RPE cells can produce and release DA. Our results indicate that 1) RPE cells can express and secrete GDNF and BDNF and protect DAergic cells against neurotxoins-induced injury; 2) RPE cells can express DDC and synthesize DA; 3) RPE cells attached to microcarriers can survive in the host striatum and produce high level GDNF and BDNF after transplantation; and 4) RPE cells transplantation produce a statistically significant improvement of AIR.

In the previous transplantation studies, microcarriers were used to increase the survival of grafted cells [21]. Indeed, microcarriers provide a substrate to which the cells can establish a basal lamina and thus create a more favorable microenvironment. Furthermore, cells attached to beads may alter the immunogenic properties of cells, which may prevent the recognition and immunological surveillance [16]. In our experiments, we used cytodex 3 which consists of a thin layer of denatured collagen chemically coupled to a matrix of cross-linked dextran, and these microcarriers facilitate the survival of transplanted RPE cells.

We demonstrate that RPE cells can provide trophic effect on DAergic cells, which may be one of the possible mechanisms underlying RPE cell therapy. Previous studies had showed that RPE cells expressed several neurotrophic factors such as PEDF, PDGF, EGF, and VEGF [11]. Our results elucidate that RPE cells can secrete BDNF and GDNF and these two factors play important role in the neurotrophic effects of RPE cells. Although RPE cells can express PEDF, it accounts for only a portion of the neurotrophic effect [22]. In this study we demonstrate that GDNF and BDNF in RPE-CM contribute for the most part of trophic effect. We also demonstrate that GDNF and BDNF are expressed by grafted RPE cells.

Besides the neurotrophic effect of RPE cells, we document that RPE cells can express DDC and produce DA. L-dopa is a precursor of DA, and can be synthesized by RPE cells as an intermediate product of melanin [23]. DDC, an enzyme to convert L-dopa to DA, is found in the RPE cells in our study. However, the depolarization-induced DA release is not detected in the cells, indicating that the DA release machinery as seen in most excitable cells is not present in the RPE cells. It's possible that RPE cells may have other mechanism to transfer DA throughout the membrane. Previous report by Dalpiaz et al [24] showed that DA could permeate the membrane of RPE cells, and this permeation seems to be mediated by organic cation transporter 3 [25]. The ability of DA synthesis in RPE cells suggests RPE cells transplantation may be one of the advantages for the cell replacement therapy to treat advanced PD patients.

## Conclusion

RPE cells not only replenish L-dopa as elucidated by previous study, but can also synthesize DA and neurotrophic factors which protect the intrinsic neurons after transplantation. These findings make this cell replacement a more viable and promising therapy for PD.

## Competing interests

The authors declare that they have no competing interests.

## Authors' contributions

The studies were designed by MM and WL and were performed by MM, XL, and XF. Human RPE cells were separated and cultured by QG. DY, LL and SC gave advises on the work and helped in the interpretation of the data. WL supervised all the work and wrote the paper together with MM. All authors read and approved the final manuscript.
